# Asymptomatic *Plasmodium vivax* parasitaemia in the low-transmission setting: the role for a population-based transmission-blocking vaccine for malaria elimination

**DOI:** 10.1186/s12936-018-2243-3

**Published:** 2018-02-21

**Authors:** Thomas C. S. Martin, Joseph M. Vinetz

**Affiliations:** Division of Infectious Diseases, Department of Medicine, University of California San Diego, La Jolla, CA USA

**Keywords:** *Plasmodium vivax*, Transmission blocking vaccine, Premonition, Asymptomatic parasitaemia

## Abstract

*Plasmodium vivax* remains an important cause of morbidity and mortality across the Americas, Horn of Africa, East and South East Asia. Control of transmission has been hampered by emergence of chloroquine resistance and several intrinsic characteristics of infection including asymptomatic carriage, challenges with diagnosis, difficulty eradicating the carrier state and early gametocyte appearance. Complex human-parasite-vector immunological interactions may facilitate onward infection of mosquitoes. Given these challenges, new therapies are being explored including the development of transmission to mosquito blocking vaccines. Herein, the case supporting the need for transmission-blocking vaccines to augment control of *P. vivax* parasite transmission and explore factors that are limiting eradication efforts is discussed.

## Background

At a time when malaria eradication is advocated as the ultimate goal of malaria control strategies worldwide, the mechanisms by which malaria remains endemic in the low-transmission setting remain unclear, and hence novel approaches to regional malaria elimination are difficult to approach. The 2017 World Malaria Report notes that in the Americas, 12 of the 18 malaria-endemic countries are projected to reduce malaria case incidence by at least 40% by 2020, but that malaria cases increased in 4 (Nicaragua, Panama, Peru and Venezuela) in the period 2010–2016 [1]. Brazil and Venezuela accounted for about two-thirds of reported cases in the Americas, and the increase in Peruvian malaria cases after 2009 has reversed gains since 2000 [1]. These trends yet again demonstrate the fragility of malaria control and elimination efforts that are subject to the vagaries of political and socio-demographic trends.

In areas of relatively low levels of transmission, such as the Amazon basin, *Plasmodium vivax* causes considerable morbidity, similar to many other regions such as those in the Horn of Africa, south and southeast Asia [1]. The geographic range of *P. vivax* malaria is broader than that of *Plasmodium falciparum* malaria, yet both declined considerably during the 20th century, especially over the past decade. Given continued global population growth, the global population at risk for malaria now estimated at 3.5 billion people [1]. The WHO estimates that from this pool the current number of new *P. vivax* infections is in the range 6.6–10.8 million cases per year with roughly 3000 deaths; however, previous studies have suggested that this is a dramatic underestimate with annual global cases of vivax malaria possibly being as high as 391 million [1, 2].

To date the most effective approaches to malaria control have been through direct and indirect interventions against the mosquito vector. Vector control in the 20th century though improvements in sanitation and the introduction of dichlorodiphenyltrichloroethane (DDT) in the 1940s (but stopped in the 1970s–1980s in South America [3–5]), combined with effective chemotherapy and chemoprevention dramatically reduced the reach of *P. vivax* malaria. However, several key host and parasite characteristics of *P. vivax* malaria have enabled this parasite to maintain endemicity, including the emergence of infectious gametocytes at the very onset of parasitaemia, the development of hypnozoites as latent liver infections in humans that later relapse, and asymptomatic parasitaemia [6–13]. Efforts to reduce the impact of endemic *P. vivax* have not established an effective way to manage ongoing transmission through relapsed infections and asymptomatic infections, especially as chloroquine-resistant *P. vivax* has spread, particularly in the Asia–Pacific region [10, 14]. Potential interventions to reduce transmission from both latently and subclinically low-level parasitaemic individuals include mass drug administration or vaccination. Given these considerations new conceptual approaches and tools to advance malaria control measures from control to elimination must still be advanced. Here the parasite and host characteristics that underlie the maintenance of regional *P. vivax* hypoendemicity, from which potential interventions, particularly transmission-blocking vaccine approaches, emerge as promising strategies are considered.

## *Plasmodium vivax* life cycle with regard to transmission-blocking vaccine development

Several aspects of the life cycle of *P. vivax* allow it to maintain endemicity but one of the most formidable is the ability to develop latent infection as hypnozoites [15]. Upon initial infection of humans, sporozoites migrate to the liver where they infect hepatocytes becoming either tissue schizonts (exoerythrocytic forms, EEFs), leading to blood stage malaria, or hypnozoites, a dormant form of the parasite that can reactivate weeks, months or years later [15, 16]. Parasite dormancy can lead to re-emergence of active infections despite active population-based vector control programmes. As the vast majority of *P. vivax* relapses are likely asymptomatic [17, 18], most do not come to medical attention and are consequently important potential sources to maintain endemicity and reintroduce *P. vivax* to human populations.

Once introduction of infection to a population occurs, diagnosis remains a challenge in some people. During the initial stages of infection, each EEF releases tens of thousands of merozoites into the blood stream, which preferentially infect reticulocytes over mature red cells. Hence infected people typically have lower parasite densities than those infected by *P. falciparum*, which invades red cells of all ages. These low level parasitaemias with *P. vivax* can lead to infections being missed by routine light microscopy or rapid diagnostic tests. This in turn means some individuals do not receive timely treatment, thus permitting ongoing transmission.

In contrast to *P. falciparum*, *P. vivax* gametocytes are sensitive to blood schizonticides as evidenced by the rapid disappearance of all parasite stages after chloroquine treatment. But, infectious gametocytes of *P. vivax* appear early in the course of infection, simultaneously with asexual forms. As a result, transmission of infection to anopheline mosquitoes can occur before adequate gametocidal therapy can be initiated. Then, after ingestion of an infection blood meal, sporogony occurs within the mosquito in which development can occur at lower temperatures than for other *Plasmodium species,* explaining its broader geographical spread and longer transmission seasons.

## Current treatment of acute, symptomatic *Plasmodium vivax* malaria and radical cure

Acute *P. vivax* malaria is conventionally treated with a blood schizonticide (i.e. chloroquine, mefloquine, atovaquone/proguanil) followed by radical cure with the 8-aminoquinoline, primaquine, initiated simultaneously. Challenges have arisen with treatment including clinically significant chloroquine-resistant *P. vivax* that has emerged in Asia, the Brazilian Amazon and Oceania [10, 19, 20]. In addition, despite treatment with primaquine, a substantial proportion of individuals (0–59% after 4–6 months) undergo relapse and can act as an ongoing reservoir for transmission [21]. The relapse rate may in part be due to non-compliance with therapy, and some studies have demonstrated improved outcomes with directly observed therapy [21, 22]. Shorter courses have also been assessed which may improve adherence as the perceived benefit of treatment falls off as symptoms subside [22]. Recent studies have suggested that 7-day courses of primaquine may be as effective as a 14-days regimen [23, 24]. Tafenoquine, a novel 8-aminoquinoline administered as a single dose, has demonstrated to be non-inferior to primaquine in clinical trials [25] and as of this writing has been submitted to the U.S. Food and Drug Administration for registration. While confirmation of radical cure is desirable, no methods in clinical use exist currently to confirm eradication of latent infection [26].

Studies of *P. vivax* relapse in endemic areas are often difficult to design and interpret because subsequent *P. vivax* infections of study subjects may result either from reinfection or relapse, which are difficult to differentiate on a molecular basis. While highly similar or identical genotypes found in sequential infections (for example as determined by microsatellites) indicate likely relapse, finding a distinct genotype does not rule out relapse because of the possibility of relapse from a different hypnozoite clone acquired at a different time by an individual [27]. One such study came from Thailand where reinfection was considered unlikely due to low prevalence or impossible due to absence of transmission. *Plasmodium vivax*-infected subjects had been transported from malaria-endemic refugee camps along the Thai border to the non-endemic region of Bangkok. The authors found that the majority of relapses were characterised by genotypic strains distinct from the prior infection [28]. This observation was felt most likely to be due to inoculation of multiple different genotypes at the original infective event, with only a few parasites becoming detectable in the blood. Alternatively, different genotypes arose from hypnozoites reactivated over time that became detectable during follow up. In either case, these authors demonstrated the inherent difficulty of establishing the cause of recurrent infection in areas with endemic transmission and consequently the difficulty of identifying the contribution of relapse to ongoing transmission.

In addition to relapse of liver-stage infection, evidence has accumulated that low level blood stage infection may contribute to ongoing transmission. Infections detectable via molecular techniques but not by light microscopy are defined as sub-patent. Studies from the Peruvian Amazon have demonstrated that as many as 14% of individuals in a cross-sectional study were PCR positive for *P. vivax* with only a quarter of these exhibiting symptoms [8]. This compares to 2.9% of individuals having parasite detected by conventional microscopy [8]. These findings are supported by other studies performed in South America suggesting that PCR detection of *P. vivax* is approximately 5–7 times more sensitive than microscopy with most individuals not reporting symptoms [6, 8, 29]. But the question arises as to whether these sub-patent infections are contributing to ongoing transmission. Alves et al. took fifteen asymptomatic individuals who were PCR positive only for *P. vivax* infection and undertook direct feeding and membrane feeding experiments with *Anopheles darlingi* with high parasitaemia symptomatic individuals as control. In total, only 1.2% of mosquitoes were infected by asymptomatic individuals compared to 22% of mosquitoes for the symptomatic carriers [7]. The authors concluded that while infection rates were lower, the much larger pool of patients and the likely longer duration of infection could mean that asymptomatic individuals represent a significant contribution to ongoing transmission.

A later study in the Peruvian Amazon followed 51 individuals with *P. vivax* infection identified through active or passive case detection [18]. Monthly follow-up included light microscopy, PCR and anti-circumsporozoite protein (CSP) antibody detection for 1 year after directly observed primaquine treatment. Twenty-nine of the 51 patients had 84 recurrent infections, of which only 26% were detectable by microscopy and just 21% reported symptoms. Sixteen of 29 patients with recurrent infection had more than one recurrence. Interestingly, about half of the 29 patients had recurrent infections that lasted 2 or more consecutive months all of which were sub-patent and asymptomatic. In three, the sub-patent infection went on to become patent. Considering all 51 patients, the overall person-infected-months was 13 per 100 person months of follow-up which equates to an average of 1.6 months per patient over the 1 year follow-up. The results suggest that *P. vivax* results in a substantial number of individuals maintaining ongoing transmission.

Since Alves et al. published landmark papers on the high prevalence of subclinical, subpatent *P. vivax* parasitaemia in Rôndonia State, Brazil [6], and the longitudinal potential contribution of such individuals to malaria transmission [7], similar observations have been made elsewhere in Amazonia [30] including Acre, Brazil [13, 31], the Loreto Department of Peru [8, 11, 12] and Colombia [32, 33].

## Asymptomatic *Plasmodium vivax* parasitaemic humans, the reservoir of endemic transmission, and mechanisms of premunition

Premunition in malaria—defined as protection against high parasitaemia and illness in the absence of completely eliminating infection—has long been known to occur [34, 35] despite what has been formally characterized as incomplete and “defective” immunity [36]; the natural history of malaria may lead to sterile immunity as well [37]. The major mechanism by which *P. falciparum* parasitaemia is controlled is widely thought to depend on the acquisition of polyclonal, protective antibodies, based on the classic study by Cohen in which the administration of immune gamma globulin was shown to lower but not eliminate *P. falciparum* parasitaemia [38].

Asymptomatic, parasitaemic individuals in endemic regions do not typically come to medical attention, hence they potentially remain infectious to mosquitoes. Asymptomatic parasitaemic individuals may have either patent or subpatent parasitaemia, and parasitaemia likely fluctuates over time and space. Hence asymptomatic parasitaemics likely move malaria parasites within the regions where they live and work [39]. The immunological mechanisms by which asymptomatic parasitaemia arises remain to be discovered, but generally speaking must be related to suppression of innate immune responses in the face of acquired immunity. Little is known about such mechanisms in *P. vivax* [40], but may relate in part to acquired antibodies against specific *P. vivax* attachment and invasion ligands (reviewed in refs. [15, 40]). Innate responses produce fever and other systemic manifestations of malaria—examples include TLR9-mediated responses to parasite DNA adsorbed to haemozoin [41] and parasite antigen-containing immune complexes [42] which produce inflammatory mediators (such as interleukin-1 and tumour necrosis factor-1) via inflammasome activation [43].

## State of malaria transmission-blocking vaccines

Transmission-blocking immunity was first demonstrated in the 1970s with an avian model based on vaccinating chickens with *Plasmodium gallinaceum* gametes [44, 45]. The discovery of Pfs25 [46], Pfs48/45 [47] and Pfs230 [48, 49] as major surface antigens of *P. falciparum* zygotes and ookinetes led to considerable efforts to develop recombinant subunit transmission blocking vaccines (TBVs) [50], including early clinical trials [51, 52]. *P. falciparum* transmission-blocking vaccine development continues [53].

On the basis of identifying the *P. vivax* orthologs of Pfs25, Pfs48/45 and Pfs230-C, TBVs based on these antigens is feasible but development of such a *P. vivax* TBV lags that of a *P. falciparum* TBV. Additional potential antigen targets of *P. vivax* TBV development remains to be explored, although the ability to obtain sexual stages of *P. vivax* (zygotes and ookinetes) to discover novel potential TBV antigens is feasible [54].

A *P. vivax* TBV is particularly attractive both because of the propensity of relapse in hypnozoite carriers and because the prevalence of asymptomatic parasitaemia in endemic regions is high. An anti-*P. vivax* TBV would either have to have sustained levels of antibodies over the time of potential relapse or populations would have to be revaccinated.

## Human infection by *Plasmodium vivax* and modulation of infectivity to mosquitoes

A key, unaddressed gap in our understanding of the natural history of *P. vivax* infection is the relationship of infection to transmissibility of parasites. The important observation that most *P. vivax* relapses are asymptomatic [18] is key to understanding how asymptomatically-infected individuals may be key reservoirs of malaria transmission over space and time.

Experimental induction of antibodies against *Plasmodium* gametes that reduce *Plasmodium* infectivity to mosquitoes has been known for decades, including those of *P. vivax*, [45, 55, 56]. Later work producing monoclonal antibodies to block transmission of *P. vivax* found that at lower concentrations, transmission of infection to mosquito may actually be enhanced [57]. The authors took monoclonal antibodies raised through inoculation of female gametes into mice and mixed the serum with the blood of patients with acute vivax infection. The authors showed that transmission was reduced to below 10% of control with a concentrated monoclonal antibody solution. With serial dilutions, however, the effect was reversed with enhancement of infectivity to approximately two–fourfold above control. The effect of serum taken from patients in convalescence demonstrated a similar effect. Among 40 patients, 3 were found to have transmission enhancement effects with one inducing a 13-fold greater than control transmission. Using convalescent serum, the authors also demonstrated a time-post-infection effect with some serum initially blocking transmission but later enhancing transmission as anti-gamete antibody titres declined. The authors postulate potential mechanisms could include enhancement of fertilization or protection from antiparasitic factors in the mosquito blood meal. Using membrane feeding assays and direct feeds to experimentally infect laboratory-bred *Anopheles tessellatus* mosquitoes, studies of *P. vivax*-infected individuals in Sri Lanka demonstrated both naturally-acquired transmission-blocking and transmission-enhancing effects at different time points in relation to acute infection [58]. While these authors found that transmission-enhancing effects were observed early in *P. vivax* infection, transmission-blocking effects occurred later after initial infection. Interpretation of the data from these studies is limited because they were based on single time point sampling and not on serial sampling, so that the natural history of transmission-modifying antibodies in individual subjects could not be ascertained.

Preliminary observations in the Peruvian Amazon in which laboratory-reared *An. darlingi* mosquitoes were infected via membrane feeding assays in the presence of serially-obtained sera (over 6 weeks) from subjects treated for acute *P. vivax*, have shown twofold increase in oocyst counts and fourfold increase in proportion of mosquitoes infected. These data further demonstrate the natural history of transmission-enhancing effects after acute *P. vivax* infection (McClean and Vinetz, manuscript in preparation).

These findings are striking in highlighting the complexity of immune response and host-parasite interaction. It also, shows the potential for complex and counter-intuitive responses to any potential vaccine dependent on immunologic response and waxing of immunity over time.

## Characteristics of a successful transmission-blocking vaccine against *Plasmodium vivax*

The concept of a vaccine blocking human infection of anopheline mosquitoes is an attractive approach to malaria control, elimination and eradication [59], but has not been robustly supported in the malaria community, at least in part because such a vaccine as a sole vaccine strategy has been considered to be “altruistic” but not of direct benefit to vaccinated individuals [46, 60–62]. Development has also been hampered by several major challenges including producing conformation of subunit vaccine proteins and the production of clinical grade material; the absence of validated standards and assays for efficacy; and the absence of a rapid pipeline of proof-of-principle human trials for testing lead candidates [63]. Based on homology with *P. falciparum* transmission-blocking vaccine candidates, the most promising *P. vivax* targets include Pvs48/45, Pvs28 and Pvs25 [64, 65].

The population impact of an effective transmission blocking vaccine is uncertain given the lack to date of phase 3 clinical trials but modelling suggests that a fivefold reduction in transmission is potentially achievable if 90% coverage could be implemented [53]. Modelling the impact of the best performing anti-Pvs25 vaccine at the time, the author predicted a 1.6-fold reduction in transmission in a 90% coverage population. Importantly, the model suggests that vaccination against multiple antigens would reduce the concentration of antibody required to achieve a given level of protection.

## Conclusion

*Plasmodium vivax* endemicity is maintained through a number of host-parasite interactions including relapse of hypnozoite stage infection, asymptomatic infection, early gametocyte appearance in the acute phase and immune interaction leading to enhanced transmission to the Anopheles vector. Disruption of these factors will be key to developing a strategy to eradicate infection and reduce the associated morbidity and mortality.

The ultimate goal of any malaria vaccine is to protect populations from malaria transmission arising from ongoing transmission and from reintroductions from movement of parasites into areas where transmission has been interrupted [66, 67]. The present discussion emphasizes that the pathway forward for a *P. vivax* transmission-blocking vaccine rests on several observations and concepts: that asymptomatic parasitaemia in highly prevalent in endemic regions; that latent hypnozoite infection is usually associated with asymptomatic relapse; that infectious gametocytes appear early in *P. vivax* infection; and that transmission-enhancing antibodies develop as an intrinsic aspect of *P. vivax* infection.
